# GOplan: an R package for animal breeding program design via integrating Gene Flow and Bayesian optimization methods

**DOI:** 10.1093/g3journal/jkae284

**Published:** 2024-12-05

**Authors:** Qianqian Huang, Lei Zhou, Yahui Xue, Heng Du, Yue Zhuo, Ruihan Mao, Yaoxin Liu, Tiantian Yan, Wanying Li, Xiaofeng Wang, Jianfeng Liu

**Affiliations:** State Key Laboratory of Animal Biotech Breeding, Frontiers Science Center for Molecular Design Breeding (MOE), College of Animal Science and Technology, China Agricultural University, Beijing 100193, China; State Key Laboratory of Animal Biotech Breeding, Frontiers Science Center for Molecular Design Breeding (MOE), College of Animal Science and Technology, China Agricultural University, Beijing 100193, China; State Key Laboratory of Animal Biotech Breeding, Frontiers Science Center for Molecular Design Breeding (MOE), College of Animal Science and Technology, China Agricultural University, Beijing 100193, China; State Key Laboratory of Animal Biotech Breeding, Frontiers Science Center for Molecular Design Breeding (MOE), College of Animal Science and Technology, China Agricultural University, Beijing 100193, China; State Key Laboratory of Animal Biotech Breeding, Frontiers Science Center for Molecular Design Breeding (MOE), College of Animal Science and Technology, China Agricultural University, Beijing 100193, China; State Key Laboratory of Animal Biotech Breeding, Frontiers Science Center for Molecular Design Breeding (MOE), College of Animal Science and Technology, China Agricultural University, Beijing 100193, China; State Key Laboratory of Animal Biotech Breeding, Frontiers Science Center for Molecular Design Breeding (MOE), College of Animal Science and Technology, China Agricultural University, Beijing 100193, China; Beijing Breeding Swine Center, Beijing 100194, China; State Key Laboratory of Animal Biotech Breeding, Frontiers Science Center for Molecular Design Breeding (MOE), College of Animal Science and Technology, China Agricultural University, Beijing 100193, China; Beijing General Station of Animal Husbandry, Beijing 100107, China; State Key Laboratory of Animal Biotech Breeding, Frontiers Science Center for Molecular Design Breeding (MOE), College of Animal Science and Technology, China Agricultural University, Beijing 100193, China

**Keywords:** breeding program evaluation, Gene Flow, Bayesian optimization

## Abstract

The design of breeding programs is crucial for maximizing economic gains. Simulation provides the most efficient measures to test these programs, as real-world trials are often costly and time-consuming. We developed GOplan, a comprehensive and user-friendly R package designed to develop animal breeding programs considering pure-bred populations and crossbreeding systems. Compared with other traditional simulators, it has mainstream crossbreeding frameworks that streamline modeling and use Gene Flow and Bayesian optimization methods to enhance breeding program efficiency. GOplan includes 3 key functions: *runCore()* to evaluate the effects of nucleus breeding programs, *runWhole()* to predict economic outcomes and the production performance of crossbreeding systems, and *runOpt()* to optimize crossbreeding structures for greater profitability. These functions support breeders in better planning and accelerating breeding goals. Additionally, the application of Bayesian optimization algorithms in this study provides valuable insights for developing new optimization algorithms in the future. The software is available at https://github.com/CAU-TeamLiuJF/GOplan.

## Teaser

This research targets animal breeders, researchers, and developers of breeding programs. Huang *et al*. developed GOplan, an easy-to-use R package designed to simulate, evaluate, and optimize breeding programs for animals like pigs, cattle, and chickens. It incorporates mainstream crossbreeding frameworks, Gene Flow methods, and Bayesian optimization to streamline crossbreeding design and improve economic outcomes. Key functions allow users to assess nucleus breeding programs, predict production performance, and optimize crossbreeding structures for profitability. GOplan simplifies complex modeling tasks and supports breeders in achieving faster genetic progress. Future updates aim to expand its functionality to plant breeding, making it even more versatile.

## Introduction

An efficient breeding program is essential for achieving long-term and substantial genetic progress in animal and plant breeding (Covarrubias-Pazaran *et al*. 2021). However, the breeding process is inherently time-consuming and costly, making it impractical to evaluate the performance of a breeding program in real-world settings. Additionally, due to the complex and dynamic nature of breeding, it is impossible to represent it through a simple or static function (Simianer *et al*. 2021). As a result, stochastic simulation has emerged as a valuable tool for managing, testing, and optimizing breeding programs.

Several simulators have been developed for simulating animal breeding programs. ZPLAN+ (Täubert *et al*. 2010; Lopez *et al*. 2016) is a well-established software built on the Gene Flow method (Hill 1974), which models genetic progress using deterministic formulae rather than stochastic simulations (Sitzenstock *et al*. 2013). However, ZPLAN+ does not account for changes in inbreeding and genetic variance. More recently developed simulators, such as AlphaSimR (Gaynor *et al*. 2021), XSim2 (Chen *et al*. 2022), and MoBPS (Pook *et al*. 2020, 2021), allow stochastic simulation of core breeding process, incorporate mainstream breeding value (BV) estimation methods, assess changes in population genetic diversity, and can be flexibly extended to design modern breeding programs (Pocrnic *et al*. 2023).

Despite these advancements, the aforementioned software lacks well-structured frameworks for crossbreeding programs. Crossbreeding, such as the 3-way crossbreeding in pigs and poultry (Sellier 1976; Dezetter *et al*. 2015; Wientjes and Calus 2017; Pérez-Baena *et al*. 2022), is a common strategy in livestock production, offering benefits such as breed complementarity (Sellier 1976), heterosis, and the creation of specialized products (Smith 1964). However, designing optimal crossbreeding programs is complex, as they involve multiple breeds with distinct breeding plans and require consideration of various interdependent factors and economic fluctuations (Henryon *et al*. 2014). Evaluating the efficiency of such programs can be challenging for breeders who may not be familiar with programming. Although some studies have compared different selection strategies via simulation (Sitzenstock *et al*. 2013; Lopez *et al*. 2016; Pocrnic *et al*. 2023), they are often limited to specific background conditions and the results are general. The benefits of each unique breeding program cannot be specifically predicted.

To address this challenge, we developed GOplan, a precompiled breeding software tailored for managing modern pure-bred and crossbred animal breeding programs. Despite evaluating the genetic progress and population diversity change of breeding programs, GOplan can effectively predict the outcomes in large-scale crossbreeding systems and optimize the population structure of the system by integrating Gene Flow methods with Bayesian optimization, avoiding massive and complex simulations. We demonstrate the application of GOplan through case studies focused on a classic 3-way crossbreeding system in pigs and provide some information on its computational efficiency. In total, GOplan can serve as an open-access and easy-to-use software to assist researchers and breeders in optimizing their unique animal breeding programs, without requiring extensive coding expertise. Detailed information on GOplan can be found on our GitHub repository (https://github.com/CAU-TeamLiuJF/GOplan).

## Methods

### Function description

**Fig. 1.**
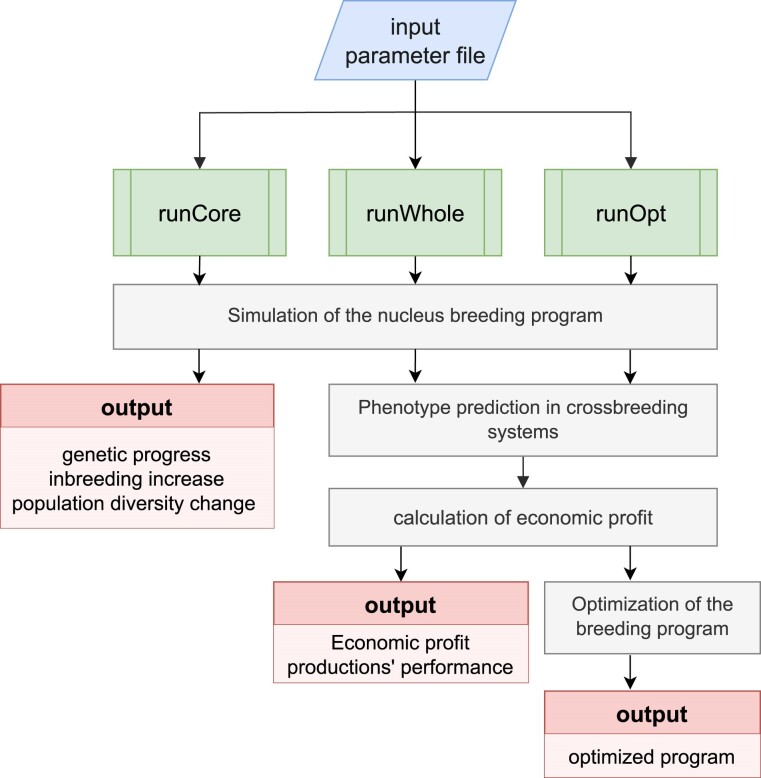
Workflow of the construction of GOplan. GOplan is directed by a single parameter file and has 3 functions with different purposes.

GOplan provides 3 core functions, namely *runCore()*, *runWhole()*, and *runOpt()*. The workflow of GOplan is outlined in Fig. 1. The *runCore()* function is tailored to evaluate different breeding programs for nucleus herds. It supports widely used BV estimation methods, including BLUP (Henderson 1975), GBLUP (Habier *et al*. 2007; VanRaden 2008), and ssGBLUP (single-step GBLUP) (Legarra *et al*. 2009), which can be executed through calling the DMU software. The *runWhole()* function can evaluate the performance of crossbred animals and the economic profitability of complex crossbreeding programs using built-in frameworks. Lastly, the *runOpt()* function employs Bayesian optimization to identify the optimal combination of parameter values that maximizes the economic profit of breeding programs.

### Simulation of the nucleus breeding program

**Fig. 2.**
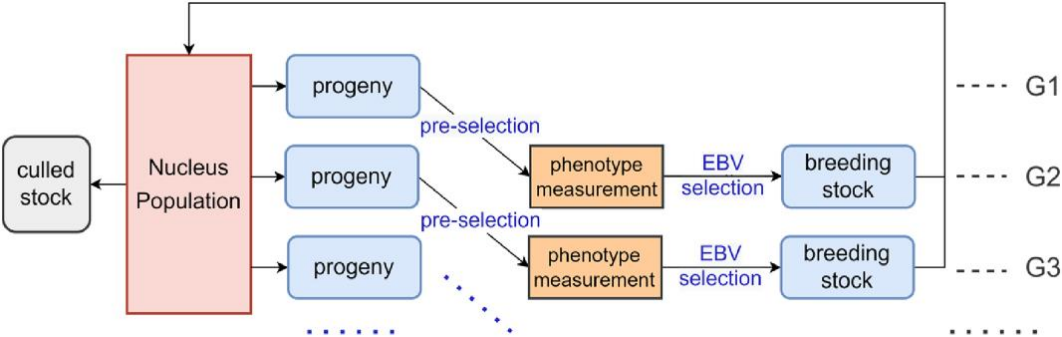
Workflow of breeding process simulation of nucleus population. $G_{i}$ means breeding season *i*. Each season, preselection for current offspring and stock culling is conducted, while the offspring born in the last year are selected to be breeding stock base on EBV.

All 3 functions need to simulate the nucleus breeding program first. The overlapping generations within the nucleus and the time required for reproduction are accounted for when simulating. In each breeding season, a subset of offspring is preselected randomly from each litter for either phenotype measurement or genotyping. These candidates are then selected based on their estimated breeding values (EBV) as future breeding stock. Concurrently, animals that have surpassed their productive lifespan are culled (Fig. 2). The breeding simulation was conducted using AlphaSimR, with the flexibility for users to customize the structure of the nucleus population by modifying the parameter file. As for function *runCore()*, only a single pure-bred population is simulated, and the genetic progress, inbreeding increase, and population diversity change after all breeding seasons are returned to assess the breeding programs' effects. However, *runWhole()* and *runOpt()* need to simulate multiple nuclei, while the genetic progress of each nucleus between consecutive breeding seasons was extracted and used to predict crossbreed animals' performance in further steps.

### Phenotype prediction in crossbreeding systems

**Fig. 3.**
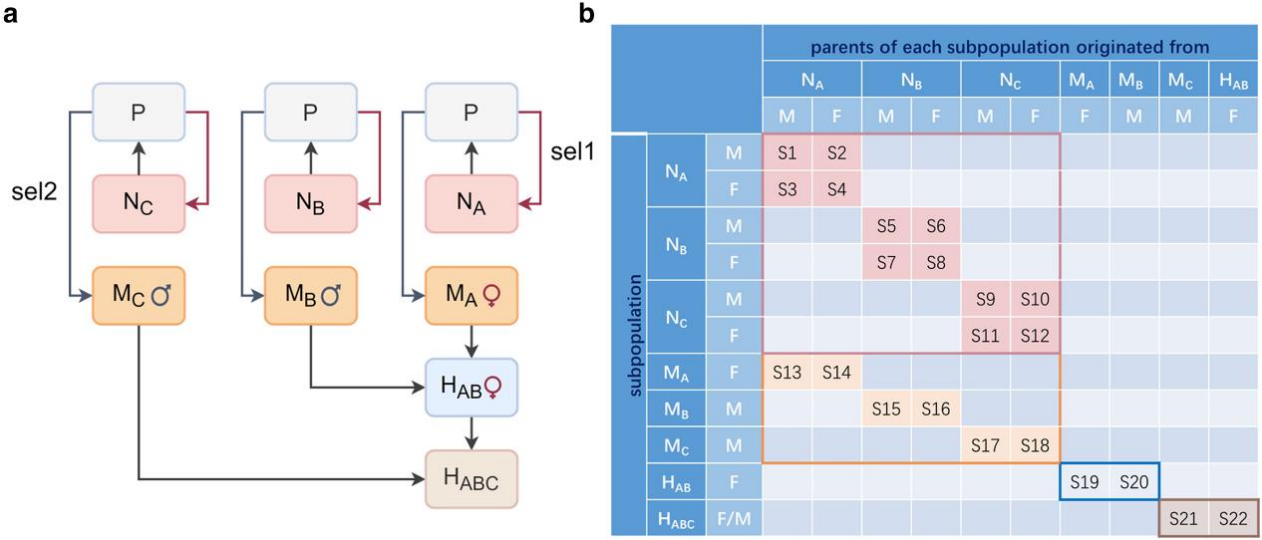
A classical 3-way crossbreeding system with multipliers for different breeds. a) The Gene Flow of 3-way crossbreeding systems, sel1: selected as breeding stock, sel2: selected as multipliers, P: the offspring of the nucleus population, N_A_, N_B_, and N_C_ are 3 nuclei of different breeds, M_A_, M_B_, and M_C_ are 3 multipliers, H_AB_ is the crossbreed population of breeds A and B, and H_ABC_ is the terminal products. b) The divided selection groups according to gender and parental origin. F and M mean females and males, respectively. S*i* means selection group *i*. For example, S1 and S2 indicate sires and dams of males in the nucleus of breed A (N_A_), respectively. Particularly, S1–18, whose parents originated from nucleus population, have permanent selection differences. In contrast, S19–22, whose parents originated from multipliers and crossbreed, have noncumulative selection differences through generations.

Given the large population sizes in crossbreeding systems, GOplan uses the Gene Flow method for phenotype prediction, significantly enhancing computational efficiency and reducing time compared with stochastic simulations. To predict the phenotype of crossbred animals, each population in the crossbreeding system is divided into distinct selection groups based on sex and ancestry. For instance, in a classic 3-way crossbreeding system with multipliers for different breeds, the population can be split into 22 selection groups (Fig. 3). GOplan supports 3 types of crossbreeding systems, namely 2-way, 3-way, and 4-way, thus covering most livestock crossbreeding scenarios.

When using the Gene Flow method, genetic progress from 1 selection group is the cumulative sum of genetic merits from all selection seasons, rather than just 1 breeding season, ensuring a comprehensive evaluation of breeding outcomes. Theoretically, the mean phenotype of the final crossbred population is the sum of the initial phenotype values and the accumulated genetic progress from all selection groups, assuming that the residual error mean is zero and no systematic effects are simulated:


$$\;\;\;\;\begin{array}{c} g_{i}=\overset{k}{\underset{\;j=1}{\sum }}\;\overset{l}{\underset{z=0}{\sum }}\;\left[sd_{ijz}\cdot \overset{l-z}{\underset{t=0}{\sum }}\;(h_{i}\cdot m_{\mathrm{jt}})\right] \end{array}\frac{n!}{r!(n-r)!}$$

where $m_{\mathrm{jt}}=R_{j}\cdot n_{t-1}+P\cdot m_{j(t-1)}$, $n_{t}=Q\cdot n_{t-1}$; $g_{i}$ is the accumulated genetic progress of the *i*th trait; *k* is the total number of pathways; *l* is the total number of breeding seasons; $sd_{ijz}$ is the pathway *j*'s genetic progress of the *i*th trait in season *z*; $h_{i}$ is the vector of realization of trait *i*'s genetic progress; $m_{\mathrm{jt}}$ is the vector of gene frequencies in the age groups of all tiers and sex groups originating from the pathway *j* in season *t*; Q is the aging matrix; $R_{j}$ is the replacement matrix of the *j*th pathway; P is the gene transmission matrix; and $n_{t}$ is the vector that demonstrates the age of breeding stock in season *t*.

### Calculation of economic profits

The economic profit was calculated using the predicted phenotypes, the number of animals, and the provided economic parameters. To ensure flexibility and adaptability to market fluctuations, costs were categorized into 6 areas: basic rearing cost per individual, maintenance costs for dams and sires, additional costs per breeding season, and phenotyping or genotyping costs per animal. Revenue was divided into 4 categories: income from production animals, revenue from culling breeding stock, and income from selling female and male breeding candidates.

For accurate profit predictions, users are required to input values for each cost and revenue component, along with the economic impact of a 1-unit trait improvement. There is no need to account for discounts, as profits are calculated separately for each season.

### Optimization of the breeding program

Bayesian optimization was employed to identify the optimal breeding program. The profit of a breeding program can be expressed as:


$$\begin{array}{c} \mu =f(x)+\epsilon \end{array}$$

where $x=[n_{H_{\mathrm{ABC}}},g_{A},\;g_{B},g_{C},\;n_{D_{A}},\;n_{D_{B}},n_{D_{C}},\;y_{M_{A}},\;y_{M_{B}},\;y_{M_{C}},\;y_{H_{\mathrm{AB}}}{]}^{\prime }$ represent the variables influencing profit; f(x) is the expected profit of the whole system under the parameter set **x**; and ϵ is the residual error.

The first 4 variables $\left(n_{H_{\mathrm{ABC}}},g_{A},\;g_{B},g_{C}\right)$ are fixed, while the remaining parameters need to be optimized. $n_{H_{\mathrm{ABC}}}$ is the expected number of production animals, and $g_{A}$, $g_{B}$, and $g_{C}$ represent the genetic progress in 3 nucleus populations. The variables $y_{M_{A}},\;y_{M_{B}},\;y_{M_{C}}$, and $y_{H_{\mathrm{AB}}}$ are the productive lifetimes of animals in the populations M_A_, M_B_, M_C_, and H_AB_, respectively. $n_{D_{A}}$, $n_{D_{B}}$, and $n_{D_{C}}$ represent the number of dams in the nucleus populations N_A_, N_B_, and N_C_, respectively.

When aiming for the maximum genetic progress in the nucleus, $g_{A},\;g_{B},g_{C}$ are usually fixed during crossbreeding program optimization. Due to the constraints of farm capacity, $n_{H_{\mathrm{ABC}}}$ is also kept constant, while breeders can adjust the female size in the nucleus populations. Consequently, the optimization problem can be expressed as:$\begin{array}{c} z^{*}=\arg\;\;\max_{z\in z}g(z) \end{array}$


$$\begin{array}{c} z^{*}=\arg\;\;\max_{z\in z}g(z) \end{array}$$

where $z=[n_{H_{\mathrm{ABC}}},g_{A},\;g_{B},g_{C}{]}^{\prime }$ and $g:z\mapsto f(z,\;n_{D_{A}},\;n_{D_{B}},\;n_{D_{C}},y_{M_{A}},$  $y_{M_{B}},\;y_{M_{C}},\;y_{H_{\mathrm{AB}}})$.

Bayesian optimization was implemented using the R package *mlrMBO* (Bischl *et al*. 2017). The optimal scheme is determined from all combinations, and the parameter file specifies the number of iterations and variable combinations sampled in each iteration.

## Results

All cases presented below are based on a 3-way pig crossbreeding system. Example parameter files used to generate these results can be found at: https://github.com/CAU-TeamLiuJF/GOplan/tree/main/inst/example_prm.

### Case 1: evaluation of the nucleus breeding program

In this case, we demonstrate the use of the *runCore()* function to simulate a nucleus population of 500 dams. The trait under evaluation is the adjusted age at 100 kg body weight (AGE). We compared the genetic outcomes of different breeding programs using combinations of 2 common factors: the number of individuals selected for phenotyping within a litter (nfam) and the productive lifetime of a nucleus sow (*Y*_d_) (Table 1).

**Table 1.** Results of different breeding programs under single-trait selection.

nfam–*Y*_d_	Relative genetic progress	Inbreeding increment	Profit/million	AGE	Decline of genetic variance
6–2	0.2429	0.1228	13.84	136.00	−8.04
6–4	0.2349	0.1133	13.95	137.63	−6.77
6–6	0.2277	0.1175	13.98	138.87	−5.65
3–2	0.2220	0.1157	11.17	139.76	−7.90
3–4	0.2152	0.1076	11.28	141.20	−6.42
3–6	0.2092	0.1025	11.30	142.26	−5.72
1–2	0.1786	0.0963	9.33	147.68	−5.98
1–4	0.1739	0.0797	9.44	148.67	−5.87
1–6	0.1707	0.0762	9.48	149.23	−4.99

The profit represents the average economic profit over all breeding seasons, and nfam–*Y*_d_ represents the variation combination of the number of individuals selected for performance testing within a litter and the productive lifetime of nucleus sow.

Among all combinations, the pairing of nfam = 6 and *Y*_d_ = 2 resulted in the highest genetic progress, though it also led to the greatest increase in inbreeding and the most significant decline in genetic variance. In contrast, while achieving lower genetic progress, the combination of nfam = 1 and *Y*_d_ = 6 resulted in the least increase in inbreeding and the smallest reduction in genetic variance, making it a suitable choice for maintaining genetic diversity.

The *runCore()* function can evaluate the effect of breeding programs based on genetic progress, inbreeding, and genetic variance, enabling breeders to select breeding programs based on their specific priorities.

### Case 2: evaluation of a crossbreeding system breeding program

In this case, we demonstrate the application of the *runWhole()* function to simulate a 3-way crossbreeding system in pigs. The initial population means for the trait AGE were 180, 170, and 160 days for 3 distinct breeds, respectively. At the beginning of the simulation, the trait value for the production animals ($H_{\mathrm{ABC}}$) was 167.5 days, calculated as half of breed C's population mean and half of the combined population means of breeds A and B.

**Fig. 4.**
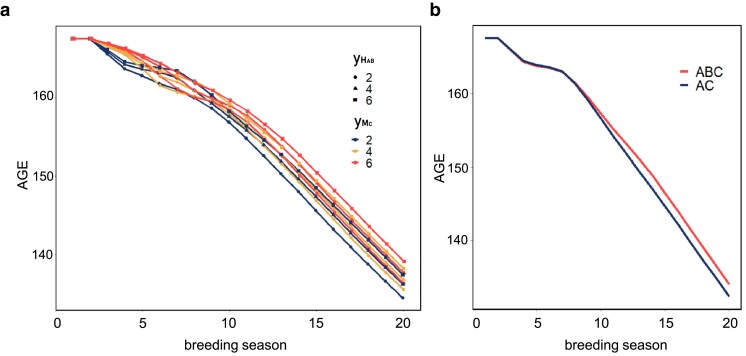
Phenotype improvement of age under different breeding systems. a) Phenotype improvement under combinations of $y_{H_{\mathrm{AB}}}$ and $y_{M_{C}}$. b) Phenotype improvement under 2 crossbreeding systems. $y_{H_{\mathrm{AB}}}$ means productive lifetime of animals in crossbreed population $H_{\mathrm{AB}}$, and $y_{M_{C}}$ means productive lifetime of animals in multiplier of breed C. AC means the crossbreeding systems with multipliers of breeds A and C, while ABC means the crossbreeding systems with multipliers of breeds A, B, and C.

We first compared the population mean of $H_{\mathrm{ABC}}$ over time, analyzing combinations of 2 variables: $y_{M_{C}}$ (the productive lifetime of the sires of production animals) and $y_{H_{\mathrm{AB}}}$ (the productive lifetime of the dams of production animals), as illustrated in Fig. 4a. The combination of $y_{M_{C}}$ = 2 and $y_{H_{\mathrm{AB}}}$ = 2 resulted in the greatest phenotypic progress, consistently showing the lowest population mean after the breeding process. In contrast, the combination $y_{M_{C}}$ = $y_{H_{\mathrm{AB}}}$ = 6 was the least successful in terms of phenotypic improvement.

Table 2 shows that the highest economic profit was achieved with $y_{M_{C}}$ = 2 and $y_{H_{\mathrm{AB}}}$ = 6, while the combination of $y_{M_{C}}$ = 6 and $y_{H_{\mathrm{AB}}}$ = 2 yielded the lowest profit. This finding aligns with expectations, as a larger $y_{H_{\mathrm{AB}}}$ leads to more production animals when the nucleus size is fixed, albeit at the cost of a longer genetic lag.

**Table 2.** Annual profit under different crossbreeding systems.

$y_{M_{C}}-y_{H_{AB}}$	Profit/million
6–6	1154.88
4–6	1155.91
6–4	767.00
2–6	1156.55
4–4	767.70
6–2	378.12
2–4	768.12
4–2	378.61
2–2	379.04

$y_{M_{C}}$ means productive lifetime of animals in multiplier of breed C, and $y_{H_{\mathrm{AB}}}$ means productive lifetime of animals in crossbreed population $H_{\mathrm{AB}}$.

In practical scenarios, it is common for farms not to set a multiplier for breed B (the sire of the dam line). To explore this, we evaluated 2 crossbreeding systems: “AC,” a 3-way system with multipliers for breeds A and C, and “ABC,” which includes multipliers for breeds A, B, and C. The results, presented in Fig. 4b, indicate that including a multiplier for breed B does not enhance phenotypic progress and, in fact, leads to a slower rate of improvement.

### Case 3: search for the optimal breeding program

The *runOpt()* function was employed to identify the optimal breeding program, targeting a specific number of production animals and a range of variable parameters. To evaluate the effectiveness of *runOpt()*, we compared its performance with a random optimization approach. This comparison involved generating an equal number of random variable combinations and assessing their performance against the results produced by *runOpt()*.

For this evaluation, we utilized the crossbreeding system established in Case 2, aiming to produce 100,000 production animals. Seven parameters were optimized: $y_{M_{A}}$ (2–6), $y_{M_{B}}$ (1–4), $y_{M_{C}}$ (1–4), $n_{H_{\mathrm{AB}}}$ (2–6), $n_{D_{A}}$ (600–3000), $n_{D_{B}}$ (300–1000), and $n_{D_{C}}$ (300–1000).

**Fig. 5.**
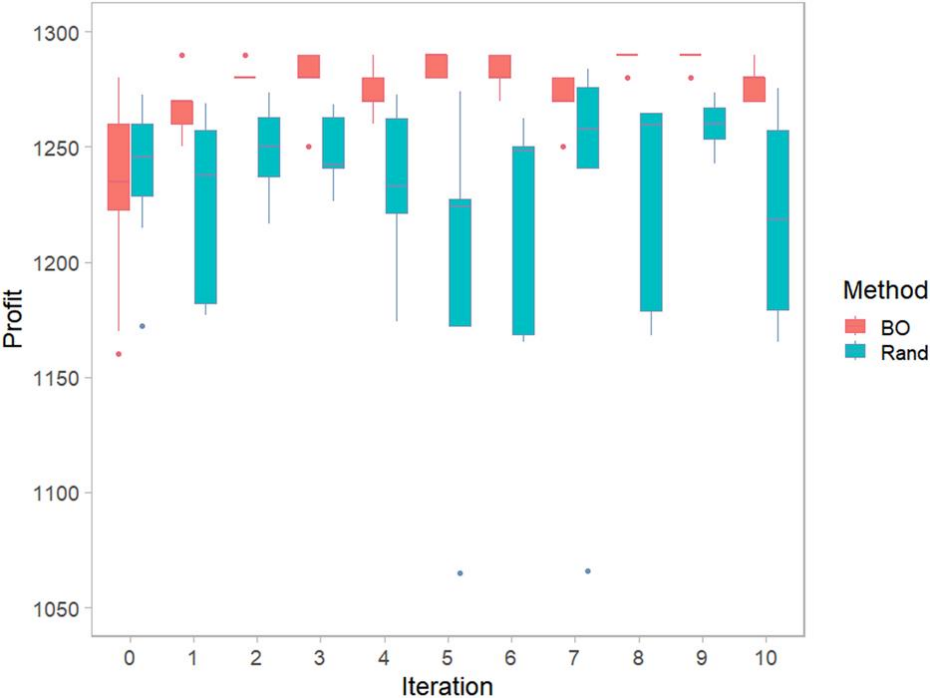
The profit of different breeding programs proposed by Bayesian optimization (BO) and random optimization (Rand). The boxplot shows the profit distribution of breeding programs explored by Bayesian optimization and random optimization in each iteration, with the *x*-axis representing the iteration rounds.

During the initial 3 rounds of searching, Bayesian optimization demonstrated gradual improvement, eventually stabilizing with minimal fluctuations. In contrast, random optimization yielded results with greater variability and less consistency. Except for the initial search outcomes, the mean profits from random optimization were generally lower compared with those obtained using Bayesian optimization (Fig. 5). Table 3 displays the top 3 optimization results obtained from ten iterations using Bayesian optimization, each with 5 sampled combinations. It is notable that the optimized schemes show comparable parameter values.

**Table 3.** Top 3 optimization results obtained by Bayesian optimization.

Number of iterations	Parameters							Profit
	$y_{M_{A}}$	$y_{M_{B}}$	$y_{M_{C}}$	$y_{H_{AB}}$	$n_{D_{A}}$	$n_{D_{B}}$	$n_{D_{C}}$	
10	3	3	4	6	2994	955	997	1290
9	5	2	4	6	3000	864	957	1290
9	3	3	4	6	2911	971	863	1290

$y_{M_{A}}$ , $y_{M_{B}}$, $y_{M_{C}}$, $n_{H_{\mathrm{AB}}}$, $n_{D_{A}}$, $n_{D_{B}}$, and $n_{D_{C}}$ are the productive lifetime of animals in population M_A_, M_B_, M_C_, H_AB_, and female size of N_A_, N_B_, and N_C_, respectively.

### Running time

We comprehensively analyzed GOplan's execution time across 3 distinct functions, with the results presented in Table 4. The running time of GOplan is influenced by several factors, including the number of repetitions, the total number of breeding seasons, the methods used for BV estimation, the volume of informative data, and the number of available processing threads. Among these, BV estimation is the most time-intensive step, particularly when employing GBLUP and ssGBLUP methods. For instance, when executing the *runCore()* function with 5 repetitions, 10 breeding seasons, and 5 processing threads, GOplan completed the task in 3.9 minutes. In contrast, the GBLUP and ssGBLUP methods extended the runtime to 23.5 minutes and 9.5 minutes, respectively. The *runOpt()* function, which explores various breeding schemes, is notably more time-consuming due to its dependence on the number of variable combinations assessed. Nevertheless, the overall running time of GOplan remains acceptable compared with the duration required for validating breeding programs in real-world scenarios.

**Table 4.** The running time of GOplan under different situations.

Scheme	Repetition	Breeding seasons	Threads	Time
*runCore()-*BLUP	5	10	1	13.6 min
*runCore()-*BLUP	5	10	5	3.9 min
*runCore()-*GBLUP	5	10	5	23.5 min
*runCore()-*ssGBLUP	5	10	5	9.5 min
*runWhole()*-BLUP	5	10	5	10.0 min
*runOpt()*	5	10	5	20.2 h

## Discussion

GOplan is a user-friendly and precompiled R package designed to assist in creating, validating, and optimizing animal breeding programs. Two main features of GOplan differ from the other simulators: firstly, it integrates classic and mainstream crossbreeding frameworks, simplifying the construction and modeling of crossbreeding systems, and then, it incorporates the Bayesian optimization method to enhance breeding program optimization. While GOplan is primarily intended for common household animals such as pigs, sheep, cattle, and chickens, it also applies to other animals with similar breeding or crossbreeding systems as detailed in this paper.

The potential applications of GOplan are demonstrated through several case studies. Firstly, the performance of nucleus-based breeding programs can be evaluated from multiple perspectives, providing comprehensive information to breeding program designers. This includes testing the effects of population structure and predicting the breeding benefits of selection indices. Secondly, the built-in crossbreeding frameworks enable users to easily model crossbreeding systems and assess their economic viability. Additionally, the Gene Flow method allows for calculating expected phenotype performance in production animals, which is useful for selecting ideal pure-bred lines for specific crossbreeding systems. Finally, Bayesian optimization helps breeders determine optimal population structure settings in crossbreeding systems to achieve improved outcomes. This method is particularly advantageous for optimizing black-box functions that are time-consuming and complex and lack clearly defined objective functions, facilitating automatic breeding program optimization (Jones *et al*. 1998). Although the current optimization parameters are limited, this feature shows potential for automating the search for optimal breeding programs and provides valuable insights for the future development of new optimization algorithms.

Despite its strengths, GOplan has some limitations. Currently, the BV estimation process relies on DMU software and breeding programs incorporating genomic mating are not yet included. Additionally, the optimization parameters available for crossbreeding programs are restricted to downstream population structures and nucleus breed sizes. Furthermore, the current application of this software is limited to animal breeding. However, plant breeding also requires similar simulation tools. Considering the differences between animal and plant breeding, we plan to develop new functions and construct frameworks for typical self-pollination and hybrid breeding plants, thereby expanding the software's applicability to a broader range of breeding scenarios.

## Data Availability

The software and material for this study are available at GOplan GitHub repository https://github.com/CAU-TeamLiuJF/GOplan.
